# iPhone now

**DOI:** 10.4103/0974-2700.41793

**Published:** 2008

**Authors:** Michael I Omori

**Affiliations:** University of South Florida Emergency Medicine Residency Program, Adult Emergency Care Center, Tampa General Hospital, Tampa, FL, USA

As of May 6, 2008, Apple Computing is planning to do a global release of it's second generation iPhone. Vodafone is projected to be the iPhone's wireless carrier in India and projected release hopefully will be later in 2008, and when you read this message. As of June 9, 2008, Steve Jobs, CEO of Apple is planning to present his keynote address at Apple's World Wide Developers Conference in San Francisco. The importance of which, is the release of third party applications, that will allow the phone to open up to the long awaited ePocrates application.

For those of you who have not experienced ePocrates, go to www.epocrates.com. This is the Internet-based medical encyclopedia, showing drug identification, doses, contraindications, and real-time pill identification. This will now be an application in iPhone. In the past, this application was available in Palm and Blackberry devices, but the superior display of the iPhone will make pill identification, in a word, sparkling.

The second generation iPhone will be thinner than the current phone, and is rumored to have an OLED lit screen, which will be brighter than the current screen and increase battery talk time. The 3G network will allow download speeds to rival broadband speed.

iPhone can be used for education where one can load teaching X-rays on the iphone and due to the iPhone's screen resolution, the pictures are remarkably clear. “YouTube” is an application that doctors should take note of. “YouTube” is an application that allows you to view downloaded video on the iPhone.

Doing a websearch on the iPhone, doctors can view medical procedures and lectures on the screen via YouTube. This service is free of charge.

EMRAP.tv is a pay application that allows you to download lectures from the staff of University of Southern California and University of California at Los Angeles.

Even the University of South Florida Emergency Medicine Residency has several Podcasts that can be downloaded for review on www.usfemresidency.com.

The future is now! Join the iPhone revolution.
